# LncRNA and mRNA expression profiles and functional networks of hyposalivation of the submandibular gland in hypertension

**DOI:** 10.1038/s41598-020-70853-x

**Published:** 2020-08-18

**Authors:** Zhu-Jun Shen, Ye-Chen Han, Yi-Ning Wang, Hong-Zhi Xie

**Affiliations:** Department of Cardiology, Peking Union Medical College Hospital, Chinese Academy of Medical Sciences and Peking Union Medical College, No. 1 Shuaifuyuan, Dongcheng District, Beijing, 1000730 China

**Keywords:** Cardiology, Molecular medicine

## Abstract

Hyposalivation is a complication of hypertension. However, little is known about the role of long non-coding RNAs (lncRNAs) in salivary glands in hypertension. This study aimed to compare the lncRNA and mRNA expression profiles between spontaneous hypertension rats (SHRs) and Wistar-Kyoto (WKY) rats through microarray analysis and apple bioinformatics methods to analyse their potential roles in hyposalivation. The differentially expressed (DE) lncRNAs and mRNAs were confirmed by quantitative real-time PCR (qRT-PCR). Compared with WKY rats, 225 DE lncRNAs and 473 DE mRNAs were identified in the SMG of SHRs. The pathway analyses of DE mRNAs showed that inflammatory mediator regulation of transient receptor potential channels was involved in hyposalivation in SHRs. Ten DE lncRNAs were chosen for further research. A coding-non-coding gene co-expression (CNC) network and competing endogenous RNA (ceRNA) network analysis revealed that the potential functions of these 10 DE lncRNAs were closely connected with the processes of the immune response. This study showed abundant DE lncRNAs and mRNAs in hypertensive SMGs. Furthermore, our results indicated strong associations between the immune response and hyposalivation and showed the potential of immune-related genes as novel and therapeutic targets for hyposalivation.

## Introduction

Hypertension is highly prevalent in the general population. Approximately one-third of adults, or approximately one billion people, are affected by hypertension. By 2025^1^, this number will increase to 16 billion. It is characterized by increased systemic arterial blood pressure (systolic blood pressure ≥ 140 mmHg and/or diastolic blood pressure ≥ 90 mmHg) and functional or organic damage to the heart^2,3^, brain, kidneys and other organs. It has been reported that Na^+^ imbalance may play an important role in the occurrence and development of hypertension. In rats with various forms of experimental and hereditary hypertension, Na^+^ reabsorption is enhanced^4^. In addition, Na^+^ transport plays an important role in regulating salivation^5^. Studies have found that hypertension should influence salivary function^6,7^. However, the molecular mechanism underlying the pathology of hyposalivation in hypertension is still unknown.

Xerostomia, a term derived from the Greek words ξηρός (xeros), meaning "dry", and στόμα (stoma), meaning "mouth", is the dryness of the oral cavity resulting from hyposalivation that is the objective finding of reduced salivary flow. A decrease in saliva secretion can increase the risk of opportunistic infection, caries, periodontal disease, denture retention decline and traumatic ulcers, which has a negative impact on the quality of life^8^. Although salivation decreases with age, the real causes of xerostomia in elderly individuals may be drug-induced hyposecretion of saliva, head and neck irradiation and systemic diseases, such as Sjogren’s syndrome, type 2 diabetes and hypertension^9^. A recent study showed that the salivary secretion of the submandibular gland (SMG) in spontaneously hypertensive rats (SHRs) was significantly reduced. The proteomic analysis of the SMG in SHRs showed that the expression of 95 proteins had changed. Specifically, aquaporin 5 and parvalbumin, related to water transport and intracellular Ca^2+^ signal transportation, have been shown to have protein expression differences^10^. McKinley et al. injected angiotensin II into the parotid gland through the carotid artery, resulting in a substantial decrease in the salivary secretion rate, which indicated that angiotensin II has a direct inhibitory effect on the parotid gland, which may be mediated by a constricting action on its vasculature or alterations in water and electrolyte transport^11^. These studies suggested that there should be a relationship between hypertension and damaged salivary function.

Long non-coding RNAs (lncRNAs), with a length of more than 200 nucleotides, are a kind of non-coding transcript for which the transcript has almost no protein coding function. LncRNAs can regulate the expression of target genes at the level of transcription and post-transcription. LncRNAs are widely involved in biological processes, such as signal transduction, molecular decoys, scaffolding and guiding ribonucleoprotein complexes^12^. Abnormally expressed lncRNAs are also associated with a variety of human diseases, such as various cancers^13^ and respiratory diseases^14,15^. An increasing number of studies have shown that lncRNAs are involved in the development of cardiovascular disease. Multiple lncRNAs are involved in the development of hypertension^16^, and lncRNA-AK094457 is considered to be a key regulator of blood pressure and endothelial function. It can increase angiotensin-II-induced vascular dysfunction by inhibiting PPARγ^17^. LncRNA-AK098656 regulates the biological function of vascular smooth muscle cells and promotes hypertension^18^. LncRNA-TUG1/miRNA-145-5p/FGF10 promotes the proliferation and migration of vascular smooth muscle cells in hypertension by activating the Wnt/β-catenin pathway^19^. However, few studies have reported the regulatory roles of lncRNAs in hypertension and the SMG^18^.

In this study, we used SHRs as the model to measure the salivary secretion of the SMG. Microarray analyses were used to investigate the differentially expressed (DE) lncRNAs and DE mRNAs in the SMG. A coding-non-coding gene co-expression (CNC) network and the competing endogenous RNA (ceRNA) network were constructed for several DE lncRNAs to predict the key genes involved in hyposalivation in hypertension. Finally, Gene Ontology (GO) and Kyoto Encyclopedia of Genes and Genomes (KEGG) analyses of the predicted target genes were carried out to identify the related pathways. The findings of this study offer new insights into the role of lncRNAs in salivary gland function in patients with hypertension.

## Materials and methods

### Animals

Five male 14-week-old SHRs weighing 200–400 g were purchased from Charles River Laboratories (Beijing, China). The same number of Wistar-Kyoto (WKY) rats, matched in age and weight, were used as the control group. Rats were kept in a humidity- and temperature (24 °C)-controlled animal room with a light:dark cycle (12:12 h) in different cages for each group. The animals were allowed free access to the same diet. The animal experiments were approved by the Experimental Animal Welfare Ethics Branch and the Biomedical Ethics Committee of Peking University (Beijing, China). The experimental procedure conformed to the Guide for the Care and Use of Laboratory Animals (NIH Publication No. 85-23, revised 1996). All animal studies were reported in accordance with the ARRIVE guidelines (Kilkenny et al. 2010)^20^. The SMGs were carefully removed from the anaesthetized rats, frozen in liquid nitrogen and stored at − 80 °C.

### Measurement of stimulated salivary flow of SMGs

The rats were fasted randomly for 12 h. After anaesthesia with an intraperitoneal injection of chloral hydrate (0.4 g/kg body weight), the SMG duct was isolated and inserted into a capillary tube. The volume of saliva was measured 10 min after the injection of pilocarpine (10 μg/kg body weight)^21^.

### Histological evaluation

SMG tissue blocks were placed in 4% neutral paraformaldehyde for 24 h, followed by routine paraffin embedding and the preparation of 5-µm slices. The sections were deparaffinized and hydrated and then stained with haematoxylin and eosin (H&E). After dewaxing and staining with H&E, pathological changes in SMG tissue were observed under a light microscope (Q550CW, Leica, Manheim, Germany) (n = 5). Five fields of each section were randomly chosen.

### Microarray gene expression analysis

All microarray hybridizations and analyses were carried out by KangChen Biotech (Shanghai, China). Total RNA from the SMG tissue was quantified using the NanoDrop ND-1000. RNA integrity was assessed by standard denaturing agarose gel electrophoresis. Sample labelling and microarray hybridization were based on the Agilent One-Color Microarray-Based Gene Expression Analysis protocol (Agilent Technology) with minor modifications. Agilent Feature Extraction software (version 11.0.1.1) was used to analyse the acquired array images. Quantile normalization and subsequent data processing were performed using the GeneSpring GX v12.1 software package (Agilent Technologies). After quantile normalization of the raw data, lncRNAs and mRNAs for which at least 5 out of 10 samples had flags of Present or Marginal (“All Targets Value”) were chosen for further data analysis. LncRNAs and mRNAs that were differentially expressed between the two groups with statistical significance were identified through *P*/false discovery rate (FDR) filtering. The DE lncRNAs and DE mRNAs were identified by volcano plot filtration with a twofold threshold and *P* < 0.05. Hierarchical clustering was carried out to show the distinguishable lncRNA and mRNA expression patterns among samples^22–24^. The GEO accession number of the dataset of this study is GSE144438.

### Quantitative real-time PCR (qRT-PCR) analysis

TRIzol reagent was used to isolate the total RNA of SMGs tissues according to the manufacturer's protocol. The RevertAid First Strand cDNA Synthesis Kit (Promega, Madison, WI, USA) was used to synthesize cDNA from 2 μg of total RNA. qRT-PCR was carried out by using FastStart universal SYBR Green Master (ROX) on a BioRad CFX96 system (Thermo Fisher Scientific). β-Actin served as an endogenous control. The sequences of the primers are listed in Supplementary Tables S1 and S2.

### Gene ontology and pathway analyses

GO analysis was used to explore the biological roles of DE mRNAs, including three domains: molecular functions (MFs), biological processes (BPs), and cellular components (CCs). Fisher’s exact test was used to determine if the overlap between the DE list and the GO annotation list was greater than that which would be expected to occur by accident. KEGG pathway analysis was carried out to determine the significantly enriched pathways in the DE lncRNA host genes. Fisher’s *P-*value denotes the significance of the pathways associated with the conditions. Lower *P-*values indicate more significant pathways (the cut off value of *P-*value is 0.05).

### Coding-non-coding co-expression network analysis

To reveal the potential regulatory relationships between lncRNAs and mRNAs, a hybrid hierarchical clustering algorithm was used to identify the co-expression of all DE lncRNAs and DE mRNAs. The lncRNA–mRNA pairs were identified by PCC values of no less than 0.95.

### lncRNA–miRNA–mRNA regulatory network construction

The potential miRNA response elements (MREs) were identified within the sequences of DE lncRNAs and DE mRNAs. We used miRanda (https://www.microrna.org/microrna/) to predict miRNA binding seed sequence sites, and the overlapping of the same miRNA binding site on both lncRNAs and mRNAs represented a lncRNA–miRNA–mRNA interaction. The ceRNA network was constructed and illustrated using Cytoscape (v3.4.0).

### Statistical analysis

Data are expressed as the mean ± the standard error of the mean, and Student's *t* test (Mann–Whitney *U*) was used to determine the difference between two groups. *P* < 0.05 was considered to indicate a statistically significant difference. Statistical analyses were carried out with the GraphPad Prism v5.0 package (GraphPad Software, Inc., La Jolla, CA, USA).

## Results

### Fluid secretion from the submandibular glands was decreased in the SHRs

To study whether the secretion of the SMGs in SHRs was affected, saliva was collected in vivo from SHRs and from WKY rats used as controls. The total volumes of secreted saliva in response to 10 μg/g pilocarpine were measured. Compared with WKY rats, the stimulated salivary flow rate of the SMGs was significantly decreased in SHRs (Supplementary Fig. S1). These data showed that the secretory function of the SMG in SHRs was damaged. H&E staining showed no local lymphocyte infiltrates in the SHRs. Observation under the optical microscope revealed no obvious differences between the WKY rats and SHRs (Supplementary Fig. S2).

### Global profiling of lncRNAs and mRNAs in SHRs and WKY rats

**Figure 1:**
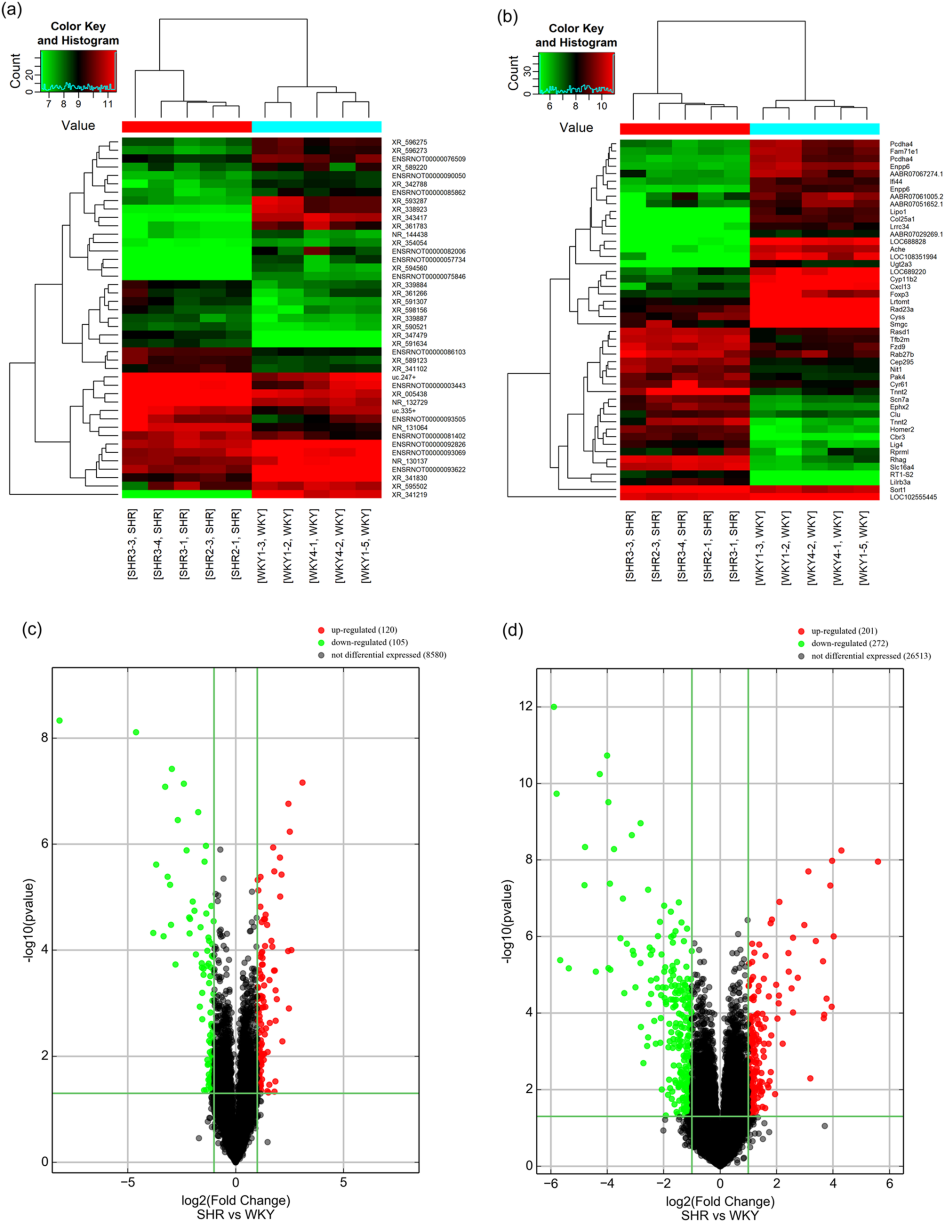
The expression profiling changes of lncRNAs and mRNAs in the submandibular glands of rats. Heatmaps showing the expression profiles of (**a**) lncRNAs and (**b**) mRNAs; volcano plots presenting differences in the expression of (**c**) lncRNAs and (**d**) mRNAs between the SHRs and WKY rats. Values plotted on the x- and y-axes represent the averaged normalized signal values of each group (log2-scaled).

We identified 225 lncRNAs (120 upregulated lncRNAs, 105 downregulated lncRNAs) and 473 mRNAs (201 upregulated mRNAs, 272 downregulated mRNAs) that were differentially expressed with a fold change ≥ 2.0 and a *P-*value < 0.05. The DE lncRNAs and DE mRNAs between the control group and SHRs are displayed in the volcano plot and heatmap. The heatmap shows the top 50 DE lncRNAs and DE mRNAs based on fold change (Fig. 1a,b). The volcanic plot shows all the DE lncRNAs and DE mRNAs (Fig. 1c,d). The top 10 upregulated and downregulated DE lncRNAs are listed in Supplementary Table S3. The top 10 upregulated and downregulated DE mRNAs are listed in Supplementary Table S4.

### Validation of DE lncRNA and mRNA expression levels

**Figure 2:**
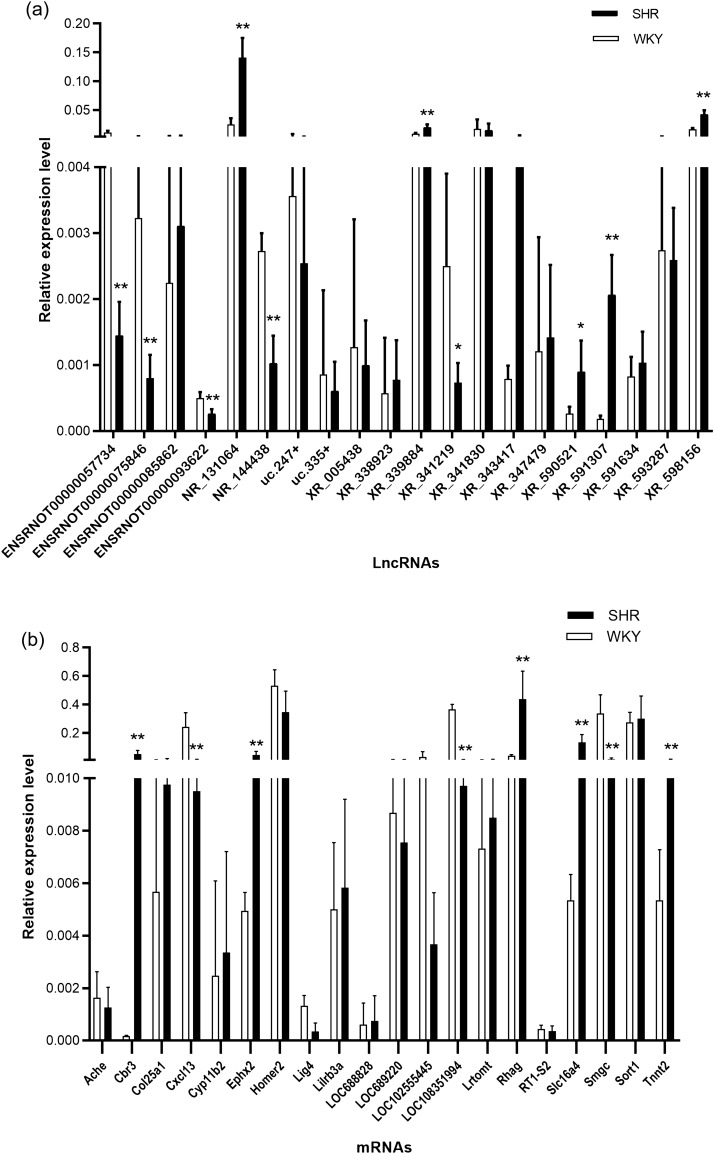
Validation of differentially expressed lncRNAs and mRNAs. (**a**) Differentially expressed lncRNAs were confirmed by qRT-PCR. (**b**) Differentially expressed mRNAs were confirmed by qRT-PCR. N = 5/group, *P < 0.05, **P < 0.01.

The expression of 20 selected DE lncRNAs was validated by qRT-PCR (Fig. 2a), 10 of which were consistent with those of the microarray analysis, as follows: 5 downregulated lncRNAs (ENSRNOT00000057734, ENSRNOT00000075846, ENSRNOT00000093622, NR_144438, XR_341219) and 5 upregulated lncRNAs (NR_131064, XR_339884, XR_590521, XR_591307, XR_598156). The expression of 20 selected DE mRNAs was validated by qRT-PCR (Fig. 2b), 8 of which were consistent with those of the microarray analysis, as follows: 3 downregulated mRNAs (Cxcl13, LOC108351994 and Smgc) and 5 upregulated mRNAs (Cbr3, Ephx2, Rhag, Slc16a4 and Tnnt2).

### GO and KEGG analyses of DE mRNAs

**Figure 3:**
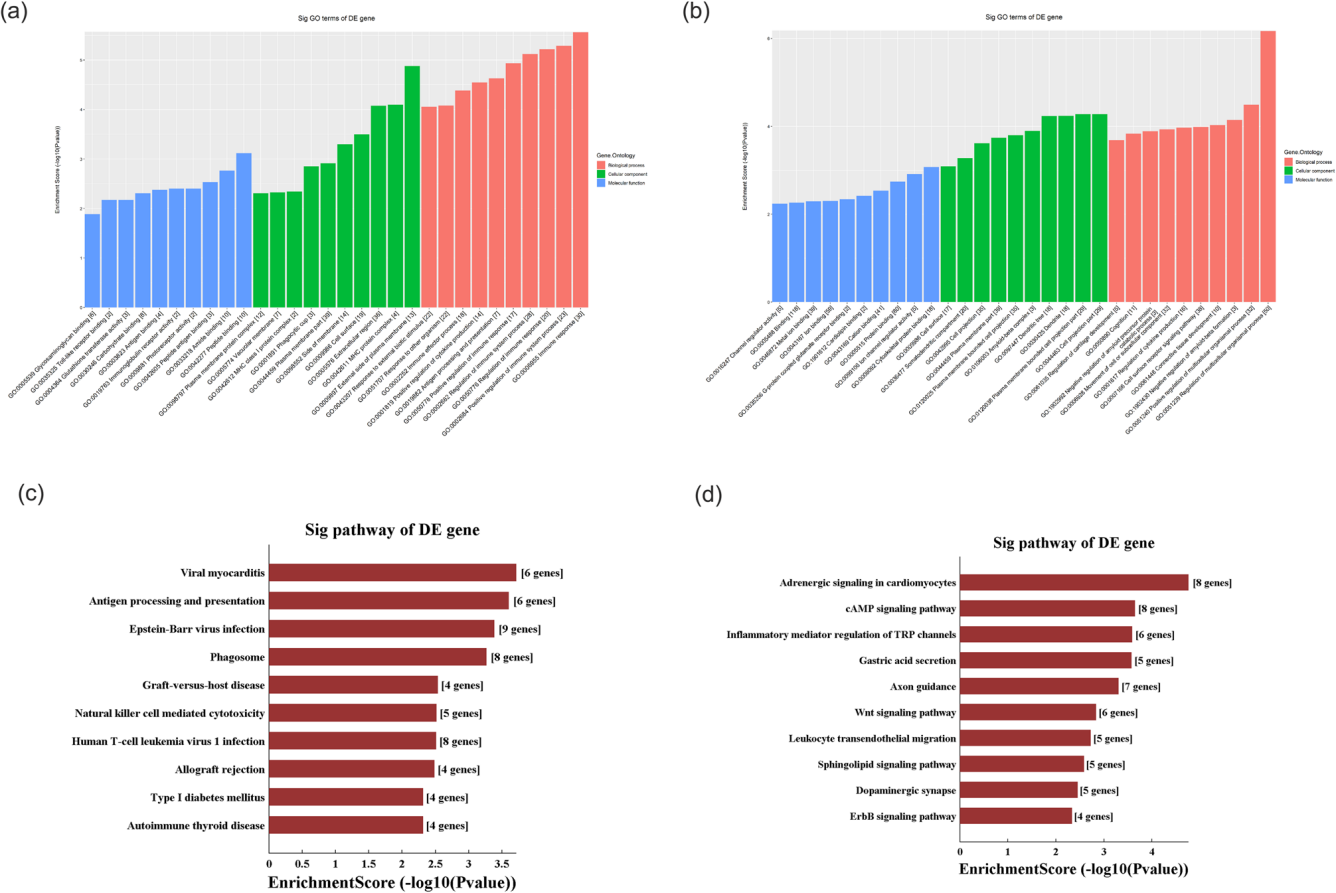
Gene Ontology and Kyoto Encyclopedia of Genes and Genomes pathway analyses analysis of differentially expressed mRNAs. (**a**) Top 10 terms from downregulated GO analysis. (**b**) Top 10 terms from upregulated GO analysis. (**c**) Downregulated mRNAs were clustered by KEGG analysis. (**d**) Upregulated mRNAs were clustered by KEGG analysis. *GO* Gene Ontology, *KEGG* Kyoto Encyclopedia of Genes and Genomes.

We performed GO and KEGG analyses of DE mRNAs. The top 10 GO terms related to BPs, CCs and MFs are shown in Fig. 3a,b. We found that the most upregulated DE mRNAs were involved in the regulation of multicellular organismal processes (an BP), cell projection part (an CC) and cytoskeletal protein binding (a MF); the most downregulated DE mRNAs were involved in the immune response (an BP), the external side of the plasma membrane (an CC) and peptide binding (a MF).

KEGG pathway analysis showed that there were 50 pathways involved in upregulated DE mRNAs and 32 pathways involved in downregulated DE mRNAs. In the upregulated DE mRNAs, the cAMP signalling pathway, inflammatory mediator regulation of TRP channels, Wnt signalling pathway and other pathways were much more highly enriched. In the downregulated DE mRNAs, antigen processing and presentation, natural killer cell-mediated cytotoxicity, human T cell leukaemia virus 1 infection and other pathways were much more highly enriched. The top 10 enriched pathways for upregulated and downregulated DE mRNAs are listed in Fig. 3c,d.

### Construction of the CNC network

**Figure 4:**
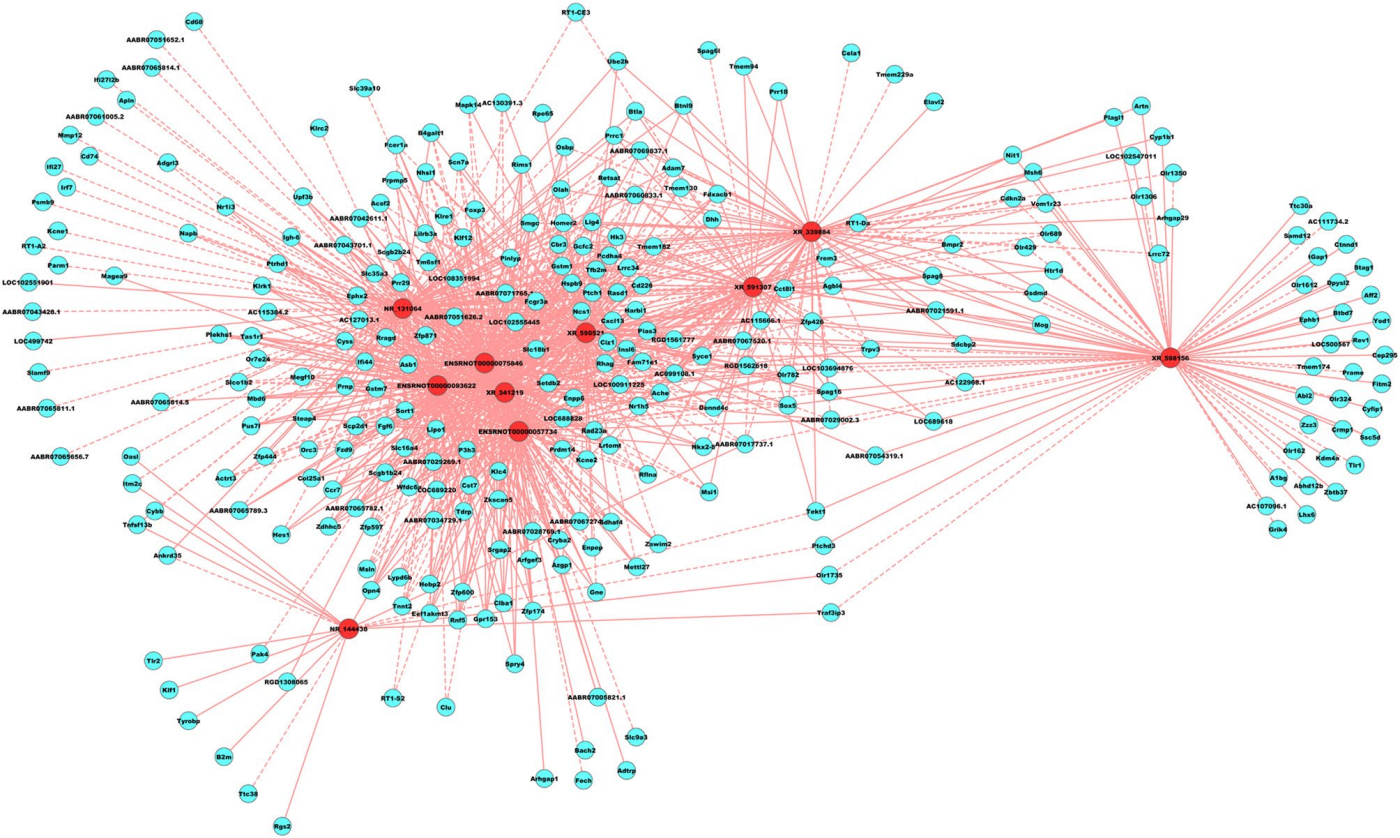
Coding-non-coding co-expression network analysis. Red nodes are lncRNAs; blue nodes are mRNAs. Positive correlation is a solid line, negative correlation is a dashed line.

Correlation coefficients were calculated between the normalized data of 10 DE lncRNAs validated by qRT-PCR and the normalized data of DE mRNAs, and those with Pearson’s correlation coefficient (PCC) values greater than 0.95, *P-*value ≤ 0.05 and FDR ≤ 1 (total 1,094) were selected to construct the CNC network. ENSRNOT00000057734 was correlated with 121 mRNAs, ENSRNOT00000075846 was correlated with 150 mRNAs, ENSRNOT00000093622 was correlated with 128 mRNAs, NR_131064 was correlated with 141 mRNAs, NR_144438 was correlated with 17 mRNAs, XR_339884 was correlated with 86 mRNAs, XR_341219 was correlated with 159 mRNAs, XR_590521 was correlated with 133 mRNAs, XR_591307 was correlated with 82 mRNAs, and XR_598156 was correlated with 77 mRNAs. The CNC network plot is shown in Fig. 4.

**Figure 5:**
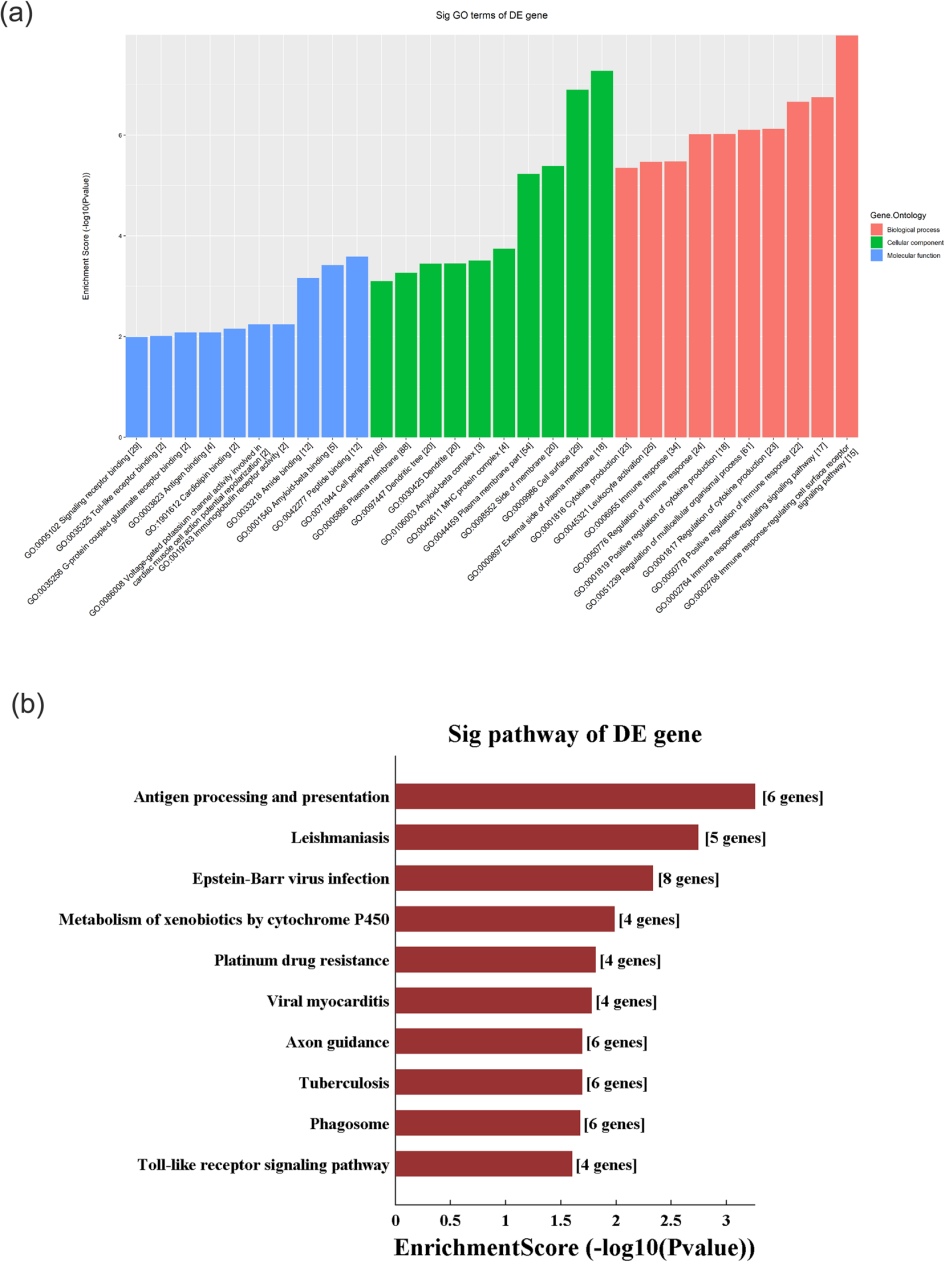
Gene Ontology and Kyoto Encyclopedia of Genes and Genomes pathway analyses based on the CNC network. (**a**) GO analysis, (**b**) KEGG pathway analysis. *GO* Gene Ontology, *KEGG* Kyoto Encyclopedia of Genes and Genomes.

Then, we performed GO and KEGG analyses based on the predicted target genes. GO analysis showed that the top two enriched BPs were the immune-response-regulating cell surface receptor signalling pathway (GO:002768) and the immune-response-regulating signalling pathway (G0002764). The top two enriched CCs were the external side of the plasma membrane (GO:0009897) and the cell surface (GO:0009986), and the top two enriched MFs were peptide binding (GO:0042277) and amyloid-beta binding (GO:0001540) (Fig. 5a). KEGG pathway analysis found that the main pathways were antigen processing and presentation, leishmaniasis and Epstein–Barr virus infection (Fig. 5b).

### Construction of the ceRNA network

**Figure 6:**
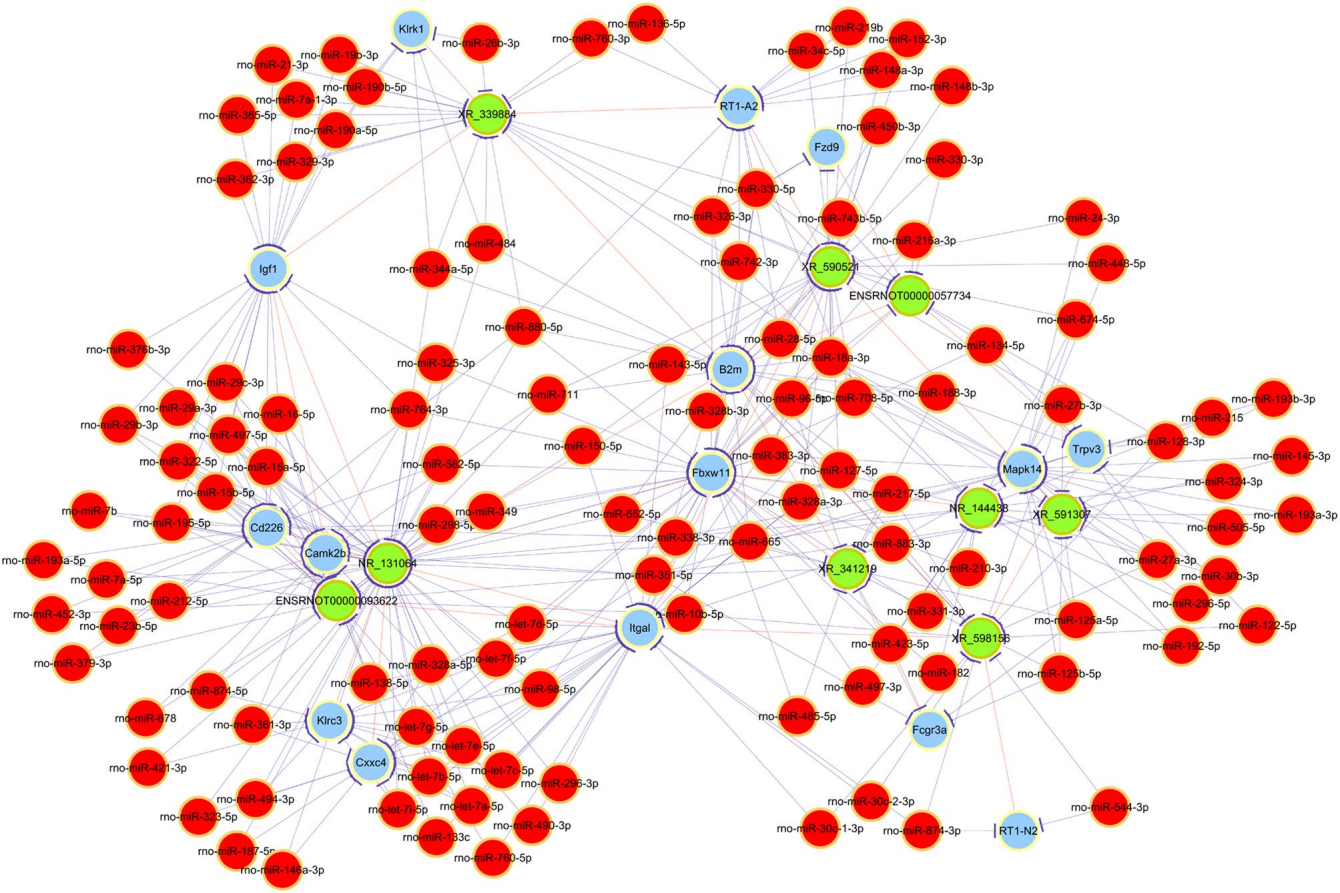
Competing endogenous RNA network analysis. Red circles represent miRNAs, blue circles represent mRNAs, and green circles represent lncRNAs.

The 10 DE lncRNAs validated by qRT-PCR, combined with DE mRNAs, were used to perform the ceRNA analysis. The number of predicted miRNA-IDs was limited to 1,000, and the predicted target genes were then used to perform KEGG and GO analyses. KEGG analysis revealed a total of 25 enriched pathways. We selected the top 5 enrichment score pathways and used the DE mRNAs in these pathways to construct the ceRNA network. The 5 pathways are as follows: antigen processing and presentation, inflammatory mediator regulation of TRP channels, the Wnt signalling pathway, cell adhesion molecules, and natural killer cell-mediated cytotoxicity (Fig. 6).

**Figure 7:**
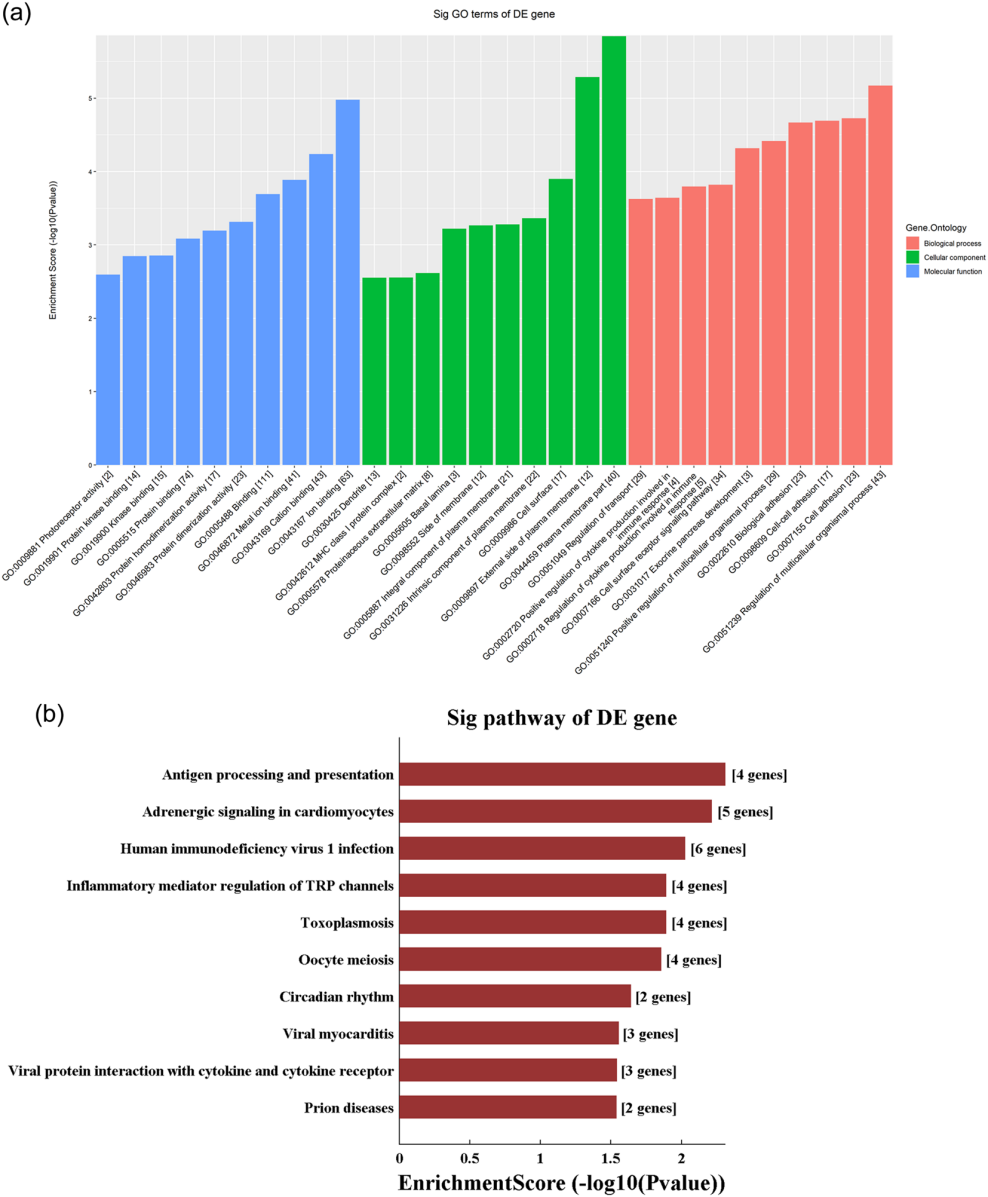
Gene Ontology and Kyoto Encyclopedia of Genes and Genomes pathway analyses based on the ceRNA network. (**a**) GO analysis, (**b**) KEGG pathway analyses. *GO* Gene Ontology, *KEGG* Kyoto Encyclopedia of Genes and Genomes.

GO analysis showed that the top two enriched BPs were the regulation of multicellular organismal processes (GO:0051239) and cell adhesion (G0007155), the top two enriched CCs were plasma membrane part (GO:0044459) and the external side of the plasma membrane (GO:0009897), and the top two enriched MFs were ion binding (GO:0043167) and cation binding (GO:0043169) (Fig. 7a). KEGG pathway analysis found that the main pathways were antigen processing and presentation, adrenergic signalling in cardiomyocytes and human immunodeficiency virus 1 infection (Fig. 7b).

**Figure 8:**
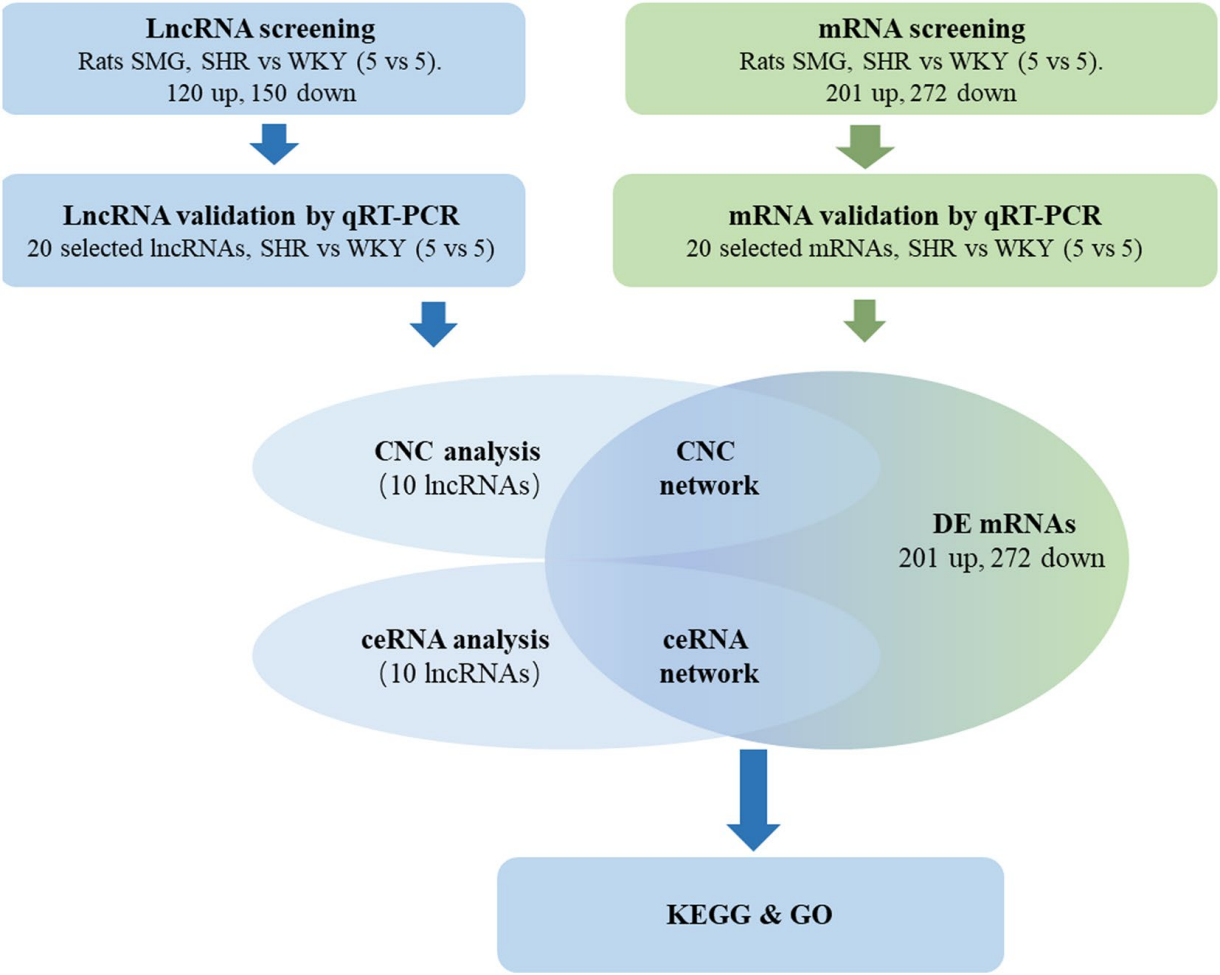
Flowchart of data collection.

The flowchart of data collection and method implementation show in Fig. 8.

## Discussion

SHRs were used in this manuscript as an animal model of essential hypertension to study the regulatory mechanism of reduced secretion of the SMG in hypertension. High-throughput sequencing of SMG lncRNAs and mRNAs from SHRs showed that there were 120 upregulated and 105 downregulated lncRNAs and 201 upregulated and 272 downregulated mRNAs compared with WKY rats.

First, GO and KEGG analyses of DE mRNAs were performed. KEGG analysis of the upregulated DE mRNA showed a higher degree of enrichment for inflammatory mediator regulation of TRP (transient receptor potential) channels (enrichment score: 3.583636). Transient receptor potential proteins have six transmembrane domains and act as ion channels with high Ca^2+^ permeability. TRP channels are composed of six subfamilies, including TRPC, TRPV, TRPM, TRPML, TRPP, and TRPA, in mammals^25^. An increasing number of studies have been carried out under the condition of various diseases, which highlights the role of Ca^2+^ signal transduction in the occurrence and development of diseases. Some of these Ca^2+^ entry channels are members of the TRP family. The secretion of fluid by the salivary glands can be stimulated by the activation of specific receptors on the cytoplasmic membrane of acini and mediated by the increase in cytosolic [Ca^2+^]^26^. Zhang et al.^10^ found that TRPV4 was a basal-lateral Ca^2+^ influx pathway in submandibular gland acinar cells, and its activation stimulated fluid secretion^27^. Radiation therapy and Sjogren's syndrome both lead to salivary gland dysfunction and xerostomia. In both cases, abnormal Ca^2+^ signalling underlies the loss of fluid secretion^28^. Therefore, TRP channels may be related to salivary secretion. We found a significant decrease in salivary secretion in stimulated SHRs. KEGG analysis showed that 6 DE mRNAs participated in the inflammatory mediator regulation of TRP channels. This suggests that TRP channels may be involved in the process of decreased salivary secretion due to hypertension. It is worth noting that we found that TRP channels are subject to inflammatory mediator regulation. Hypertension is a chronic inflammatory process. The activation of the sympathetic nervous system and the renin–angiotensin–aldosterone system may activate the release of a large number of inflammatory factors and then damage vascular endothelial cells, leading to the rise of blood pressure^29^. Studies have reported that SHRs have a large number of activated lymphocytes and monocytes and release inflammatory factors. Based on our results, we speculated that the inflammatory process induced by hypertension affects the function of TRP channels, impairing Ca^2+^ signalling (aberrant Ca^2+^ signalling), which may be involved in the reduction of salivary secretion.

We identified 10 DE lncRNAs by qRT-PCR with the most significant expression differences in the SMG of SHRs, as follows: ENSRNOT00000057734, ENSRNOT00000075846, ENSRNOT00000093622, NR_144438, XR_341219, NR_131064, XR_339884, XR_590521, XR_591307, and XR_598156. We conducted in-depth analyses of these 10 lncRNAs, including CNC analysis and ceRNA analysis. Then, we performed GO and KEGG analyses according to predicted target genes to identify related molecules and pathways regulated by these DE lncRNAs. We observed an interesting phenomenon in which many of these predicted target genes were related to the immune response. The GO analysis of the CNC network revealed that the most related BP was the immune response: immune response-regulating cell surface receptor signalling pathway (enrichment score 7.97915179902155), immune response-regulating signalling pathway (enrichment score 6.75444880796142), positive regulation of the immune response (enrichment score 6.66100197807179), the regulation of cytokine production (enrichment score 6.1222877173628), and positive regulation of cytokine production (enrichment score 6.0192956725254). KEGG analysis of the CNC network showed that antigen processing and presentation were highly enriched (enrichment score 3.25377). This suggested that the 10 DE lncRNAs identified by us were involved in the regulation of immune processes. However, the pathway by which lncRNAs regulate these mRNAs needs further study.

We also carried out ceRNA analysis to establish the relationship between these 10 DE lncRNAs and DE mRNAs. GO analysis of the downstream target genes predicted by ceRNA revealed that the top BPs involved were as follows: the regulation of cytokine production involved in the immune response (3.79663529907157) and positive regulation of cytokine production involved in the immune response (3.64351452795962). KEGG analysis also showed that antigen processing and presentation were significantly enriched (enrichment. Score 2.306039). This further suggested that lncRNAs regulated these BPs through miRNAs. This provides basic data for further research in the future.

The immune system may have been a key factor in the development of hypertension. The general assumption is that the accumulation of immune cells in blood vessels (especially perivascular fat) and the kidneys, heart and brain will promote a chronic inflammatory response, thus damaging the blood pressure regulation function of these organs, leading to hypertension^30^. It has been reported that circulating IgG levels in patients with hypertension are higher than those in individuals with normal blood pressure^31,32^. The mechanism of antibodies promoting hypertension occurs through the direct activation of receptors and channels regulating vascular tension, renal sodium reabsorption and cardiac function^33^. Cytotoxic T lymphocytes may also affect blood pressure by enhancing renal reabsorption of sodium and water^34,35^. T-helper cells may directly affect the activity of cyclooxygenase in the vascular wall, thus promoting endothelial dysfunction and increasing vascular resistance^36^. Abnormal immune activation plays a pathogenic role in the development of animal and human hypertension. SHRs had many autoantibodies in addition to decreased salivation. High levels of autoantibodies against the second extracellular loop of the a1-adrenoceptor (a1-AR autoantibody, a1-AA) are found in SHRs, and this altered responsiveness is due to endothelial dysfunction and decreased NO bioavailability^37^. Chen CH et al. observed an elevation of the plasma concentration of IgA and of circulating IgA autoantibodies to single-stranded DNA, double-stranded DNA, and thyroglobulin in SHRs^38^. At a prehypertensive age (1 month), anticardiolipin antibody levels in SHRs were significantly higher than those in control Wistar rats^39^. These suggested that an abnormal immune activation occurred in SHR. Combined with our experimental results, we believed that immunotargeted therapy will become a new target and new idea to reduce the complications of hypertension, including the reduction of salivation.

The secretory function of the salivary gland is closely related to the function of the immune system. Primary Sjogren's syndrome (PSS), systemic lupus erythematosus, rheumatoid arthritis and other systemic autoimmune diseases are usually accompanied by decreased saliva secretion^40^. PSS is characterized by chronic inflammation of the salivary and lacrimal glands, respectively, followed by dry oral and ocular mucosa^41^. The epithelial cells of the exocrine organs of PSS patients showed a large amount of lymphocyte infiltration. A T cell-related proinflammatory microenvironment and subsequent chronic inflammation lead to irreparable structural modifications and the hypofunction of the target organs, including insufficient secretion of salivary glands and severe dry mouth. At the same time, this process is also regulated by ncRNAs. The upregulated lncRNA-PVT1 in CD4^+^ T cells of PSS patients can maintain the expression of Myc to control the proliferation and effector functions of CD4^+^ T cells by regulating the reprogramming of glycolysis^42^. Decreased saliva secretion can affect oral health. Clinical studies report that high blood pressure reduces saliva secretion in patients^43–45^. Saliva plays a key role in digestion, taste, cleaning, the hydration of the oral mucosa and tooth protection and is essential to maintain the dynamic balance of the oral environment. When the secretory function of the salivary gland is damaged, the decrease in salivary secretion will affect the oral microenvironment, leading to dry mouth, taste disorders, rickets, periodontal disease, and chewing and swallowing difficulties, which decrease the quality of life. In addition, insufficient saliva secretion will lead to a decrease in the oral clearance rate, a decrease in the saliva pH value and buffering ability, and a decrease in the immune defence ability. These symptoms may increase the risks of oral diseases such as cervical caries, periodontitis and oral candidiasis^46,47^. Considering the importance of oral health, we should pay ample attention to the decrease in saliva secretion caused by hypertension. This study shows that many mRNAs in the SMG of SHRs are related to the immune response, and these molecules are regulated by lncRNAs. There are obviously aberrant immune reactions that are regulated by lncRNAs in the SMG in hypertension, and these aberrant reactions provide a new idea for the treatment of hypertension complications. The 10 lncRNAs that we identified are worthy of further study.

RNA sequencing is a powerful tool that can not only decipher non-annotated transcriptional activity but also reveal the differential expression profile of RNAs underlying specific phenotypic differences. In this study, we screened the genome-wide expression profiles of lncRNAs and mRNAs in the SMG in SHRs and found that the expression of lncRNAs and mRNAs in SMG tissues was significantly different from that of the control group, and some abnormal expression of lncRNAs may play important roles in the development and progression of hyposalivation. We show for the first time that TRP mRNAs were significantly upregulated in the SMG of SHRs, which may serve as a novel therapeutic target. We also found that lncRNAs regulate immune responses, providing a new direction to control immune responses. A potential drug that regulates one key lncRNA may be useful to suppress the occurrence of hyposalivation in hypertension. In addition, some immunosuppressors can be used to improve hyposalivation. Our study had some limitations. First, the sample size of the microarray analysis used to verify the results was small. Second, the subsequent functional verification needs to be further improved.

In the past 50 years, researchers have concentrated on the role of lncRNAs in disease processes. Disease–lncRNA association inference is important in the design of specific molecular tools for human disease diagnosis, treatment, prognosis and prevention. It is difficult to quickly and efficiently study the relationship between lncRNAs and diseases by relying only on traditional biological experiments. Chen et al. developed the powerful computational model of LRLSLDA to predict potential disease-related lncRNAs based on a semi-supervised learning framework^48^. To date, dozens of computational models have been proposed for this purpose. In general, the current computational models for lncRNA function prediction could be classified into four categories: sequence alignment-based models, gene co-expression-based models, lncRNA–miRNA/mRNA/protein interaction-based models and integrative feature-based models. Computational models could be effective ways to identify potential lncRNA functions and lncRNA–disease associations, hence decreasing the time and cost of biological experiments. In our study, high-throughput sequencing was performed in the SMG, followed by bioinformatics analysis using a computer model. This can help us quickly and efficiently identify lncRNAs associated with hyposalivation in hypertension.

In summary, we constructed the expression profile of DE lncRNAs and DE mRNAs in the SMG of SHRs. mRNA profiling revealed that inflammatory mediator regulation of TRP mRNAs was significantly upregulated in the SMG of SHRs. Our CNC and ceRNA network analyses suggested that aberrant immune responses were observed in hypertensive SMGs and were regulated by 10 DE lncRNAs. Our findings further expand our understanding of the role of lncRNAs in salivary secretion. Through the construction of the regulatory networks, our study is helpful to understand their role in the pathological process of hyposalivation in hypertension. This study may provide valuable clues for further research, as the role of lncRNAs has not been fully uncovered in hyposalivation in hypertension.

## Supplementary information

Supplementary Information.

## Data Availability

The data that support the findings of this study are available in GenBank databases under accession number GSE144438*.* Addresses are as follows: https://www.ncbi.nlm.nih.gov/geo/query/acc.cgi?acc=GSE144438.
