# Supplementary material for: LncRNA and mRNA expression profiles and functional networks of hyposalivation of the submandibular gland in hypertension
Source: Sci Rep. 2020 Aug 18;10:13972. doi: 10.1038/s41598-020-70853-x (PMC7434885; doi:10.1038/s41598-020-70853-x)

Supplementary Material

LncRNA and mRNA expression profiles and functional networks of hyposalivation of the submandibular gland in hypertension

Zhu-Jun Shen[1], Ye-Chen Han[1], Yi-Ning Wang[1], Hong-Zhi Xie[1*]

Supplementary Table S1. Primers for LncRNAs validated by qRT-PCR

Supplementary Table S2. Primers for mRNAs validated by qRT-PCR

Supplementary Table S3. The detailed information of the top ten up-regulated and top ten down-regulated lncRNAs

Supplementary Table S4. The detailed information of the top ten up-regulated and top ten down-regulated mRNAs

Supplementary Figure S1. Secretory function of the submandibular gland of rats. The stimulated salivary flow rate of submandibular glands (μl/min). N=5/group, ** $P < 0.01$.

Supplementary Figure S2. Histological structure of submandibular glands in SHRs and WKY rats. (a) H&E staining of SMGs in SHRs, (b) H&E staining of SMGs in WKY rats. bar, 50μm.

Supplementary Table S1. Primers for LncRNAs validated by qRT-PCR

Gene	Forward and Reverse primer sequence
β-actin（R）	F:5'CGAGTACAACCTTCTTGCAGC3’<br>R :5’ ACCCATACCCACCATCACAC3’
XR_591634	F:5' CTTGTTAAGTGCAAGCGACATC 3’<br>R:5’ GACGCCGACATTTCCTCTAT 3’
XR_347479	F:5' AGGCTTGAAGAGTGTGAATGAG 3’<br>R:5’ CCTTGTTGGGGTAGTTGGA 3’
NR_131064	F:5' CACGGGAGAATGCCTTACCT 3’<br>R:5’ GGGTCCACAAGAAGTTGTTCTG 3’
XR_591307	F:5' CTCAGAATAAAAGCCAGGAACT 3’<br>R:5’ GTACCAAACAATAGCCAGCC 3’
uc.335+	F:5' CACTACATTAACCACTGGGACC 3’<br>R:5’ GCATTACTGAACTTGCCACATT 3’
uc.247+	F:5' TTCTTGGGGCTGAATCGTA 3’<br>R:5’ TCGTAAAGCACTGGGCAAT 3’
XR_005438	F:5' GCCACACCATCGCCAGGA 3’<br>R:5’ GGTCAAGACCAAACAATTCGGA 3’
XR_339884	F:5' CTTAGTCTTGAATGCCCTCTGA 3’<br>R:5’ CACCCGTGTTGTTATACTTGCT 3’
XR_341219	F:5' GACAGAGCCTCCGGATCTTA 3’<br>R:5’ CACTATTGAATCGTTACAACCCT3’
XR_343417	F:5' AGAGGAGGCTCCATGAGGAAGA 3’<br>R:5’ CCCAGTGTTGAAGCATCACAAG 3’
XR_338923	F:5' GGAGAAGAAACATGAAGTGACA 3’<br>R:5’ GGAAGGCTGGTCAAACAAT 3’
ENSRNOT00000057734	F:5' CATGGTAGGAGGCAACGGAT 3’<br>R:5’ CCCAAGGGCTCATTCAAATT 3’
XR_593287	F:5' ATTAGGGCTGAGGAAATACACA 3’<br>R:5’ GGCTTTTCTTGAGTGATACCC 3’
ENSRNOT00000075846	F:5' TCATCAAGTTCACCGTCATTAG 3’<br>R:5’ GACACTCACTACACAAAGCACTG 3’
XR_341830	F:5' TGTGGTCAAAATGCGTCCC 3’<br>R:5’ TCTGCGAAACTGAAATGGC 3’
ENSRNOT00000093622	F:5' CCTGAGGGAAGATTCTCTCTGA 3’<br>R:5’ GGAACTGATGGGATGTCTCG 3’
XR_590521	F:5' CAGCATAGCGGGTCAGGG 3’<br>R:5’ CACAGGTCTACGAGAGCTTGGT 3’
XR_598156	F:5' ATGAAAAGACAGCAGAAGGATT 3’<br>R:5’ TTGCCTGTAGTCTAGGTGTTGTC 3’
ENSRNOT00000085862	F:5' TAAGCAGACAGACATCACCGAA 3’<br>R:5’ TGCCCCGTGGAAATAAGAG 3’
NR_144438	F:5' TGGATTACCTTGGTGGATTTTC 3’<br>R:5’ GCTTTGCCATTCCTTACTTCAC 3’

Supplementary Table S2. Primers for mRNAs validated by qRT-PCR

Gene	Forward and **Reverse** primer sequence
β-actin（R）	F:5'CGAGTACAACCTTCTTGCAGC3’<br>R :5’ ACCCATACCCACCATCACAC3’
Lilrb3a	F:5’ GCCTTCCTCCTGTTCCTCTT 3’<br>R:5’ CTGGGTTTGACCTCTTCTGG 3’
Slc16a4	F:5’ CAATACCACAAGGCTTACCTCA3’<br>R:5’ AGAAACTGGCATATCCCAAAA 3’
Rhag	F:5’ GGGTTCATAACTTGCACGGC 3’<br>R:5’ GCTGGTTCCAGACAGGTAGC 3’
Tnnt2	F:5’ AGACTCTGATCGAGGCTCACTT3’<br>R:5’ ATTGCGAATACGCTGCTGTT3’
Cbr3	F:5’ TGCTGCCCATAATGAAACCAC 3’<br>R:5’ TCCTGTCCGCTTTCCTCTTCT 3’
Homer2	F:5’ TGTGGATGGAGCCAAGGTAAT 3’<br>R:5’ CACTGCCCGAACTTCTGTGA 3’
RT1-S2	F:5’ CTGCCTACTTGCTCCAACTGCT 3’<br>R:5’ GGTCAGGTTCTCCCCATCCC 3’
Ephx2	F:5’ AAGATCCCAAGGTCAGCAAA 3’<br>R:5’ CAACGCCTTACAGCTCCACT 3’
Lig4	F:5’ TCGGCTGGACTGGGATTTAC 3’<br>R:5’ AGTTCGTGAGGCTTGTGAGGA 3’
Sort1	F:5’ CGCCAAGCTGACCAACAATA 3’<br>R:5’ TTCCGTAATCCTCACTGCGA 3’
LOC102555445	F:5’ TCTGAAATCCTGTGGCGTCAT 3’<br>R:5’ TGCATTGCTCCCTTCGACAT 3’
LOC688828	F:5’ GGGAGTGTCCACGTTCAACA 3’<br>R:5’ GGCCCAAGACCTCTAGTCCA 3’
LOC108351994	F:5’ ATCTGTGCCTCCAATCAAGAGC 3’<br>R:5’ AGGGTCCACTTTATGGTTCTGAG 3’
Smgc	F:5’ GCTTAGGTCCCAGATCGTCAC3’<br>R:5’ CGTCTTTGTTCCCACCAGAAT3’
Ache	F:5’ CACCGTGCCTCCACATTGACT 3’<br>R:5’ CCTCCAAAGGCTTCAGGTTCA 3’
Cxcl13	F:5’ AAGTTATACGCCCTGGGAATG 3’<br>R:5’ GCCGTGTTTGTAGAGGGAAGTT 3’
Cyp11b2	F:5’ TGGCATTGTGGCAGCACTAA 3’<br>R:5’ TGTCGTGTCAACGCTCCCA 3’
Lrtomt	F:5’ GCACCCCGCTTCCTACAAT 3’<br>R:5’ TTGGGCTATGCCATCCTTG 3’
LOC689220	F:5’ TCACTGCAGTTCCTCTTCTAACCT 3’<br>R:5’ AAATTTGGTTCCAGCCACCAC 3’
Col25a1	F:5’ CACTTTGTATCCACCGTCTCAT 3’<br>R:5’ ATCTGCCCTTCAGTCCCCT 3’

Supplementary Table S3. The detailed information of the top ten up-regulated and top ten down-regulated lncRNAs

ProbeName	Transcript_ID	P-value	Fold Change	Regulation	chrom	GeneSymbol	RNAlength
ASRNV20031939V3	XR_591634	6.90648E-08	8.5	up	chr3	Dut	785
ASRNV20028475V3	XR_347479	1.73217E-07	5.4	up	chr6	LOC102548124	2270
ASRN6LNC1A100128855V3	NR_131064	3.7537E-06	4.4	up	chr6	AABR07065531.5	1915
ASRN6LNC1A100204291V3	ENSRNOT00000003443	0.029893022	3.6	up	chrX	AABR07037410.1	3257
ASRN6LNC1A100204293V3	ENSRNOT00000093505	0.046619524	3.5	up	chrX	AABR07037410.1	3261
ASRNV20031647V3	XR_591307	0.000367257	3.0	up	chr2	LOC102555028	1507
uc.335+_P1V3	uc.335+	0.048320134	2.9	up	chr4	uc.335	214
ASRNV20029903V3	XR_589123	2.60754E-05	2.6	up	chr8	LOC102553777	471
uc.247+_P1V3	uc.247+	0.027318528	2.3	up	chr1	uc.247	361
ASRNV20026778V3	XR_005438	0.00034554	2.3	up	chr17	LOC498759	911
ASRNV20027422V3	XR_341219	4.6684E-09	286.4	down	chr16	LOC102550919	803
ASRNV20027796V3	XR_343417	7.755E-09	24.5	down	chr1	LOC102551452	2762
ASRNV20027037V3	XR_338923	2.43105E-06	12.8	down	chr10	LOC102547123	671
ASRNV20029745V3	XR_361783	5.47088E-05	10.1	down	chr19	LOC102550448	409
ASRN6LNC1A100353538V3	ENSRNOT00000057734	5.84945E-06	8.2	down	chr17	Nebl	706
ASRNV20033336V3	XR_593287	3.32592E-05	8.0	down	chr7	LOC102549242	1193
ASRN6LNC1A100442038V3	ENSRNOT00000075846	3.81146E-08	7.8	down	chr12	Gtf2ird2	474
ASRN6LNC1A100002653V3	ENSRNOT00000082006	0.000186305	6.9	down	chr19	AABR07043276.1	525
ASRNV20027520V3	XR_341830	3.51668E-07	6.4	down	chr18	LOC498829	1075
ASRN6LNC1A100002801V3	ENSRNOT00000093622	7.25787E-08	5.3	down	chr7	AABR07058280.2	715

Supplementary Table S4. The detailed information of the top ten up-regulated and top ten down-regulated mRNAs

ProbeName	Transcript_ID	P-value	Fold Change	Regulation	chrom	GeneSymbol	RNAlength
ASRN6AP1B104913577V3	ENSRNOT00000087957	1.102E-08	48.3	up	chr1	Lilrb3a	2795
ASRN6AP1B100012875V3	ENSRNOT00000024455	5.65E-09	19.7	up	chr2	Slc16a4	2010
ASRN6AP1B100064748V3	ENSRNOT00000072144	9.908E-07	16.3	up	chr9	Rhag	2138
ASRN6AP1B102997504V3	ENSRNOT00000050284	6.821E-05	15.5	up	chr13	Tnnt2	870
ASRN6AP1B100000272V3	ENSRNOT00000002310	4.652E-08	15.0	up	chr11	Cbr3	1153
ASRN6AP1B100311897V3	ENSRNOT00000047682	4.187E-05	13.7	up	chr13	Tnnt2	1192
ASRN6AP1B100036164V3	ENSRNOT00000087785	1.31E-06	10.5	up	chr1	Homer2	1985
ASRN6AP1B100564544V3	ENSRNOT00000082880	1.99E-08	8.7	up	chr20	RT1-S2	485
ASRN6AP1B100012757V3	ENSRNOT00000023385	4.999E-07	7.9	up	chr15	Ephx2	2125
ASRN6AP1B100012471V3	ENSRNOT00000019615	2.704E-06	5.4	up	chr16	Lig4	3192
ASRN6AP1B100032020V3	ENSRNOT00000074622	4E-13	59.2	down	chr7	LOC102555445	276
ASRN6AP1B100049835V3	ENSRNOT00000056435	1.858E-10	55.4	down	chr8	LOC688828	848
ASRN6AP1B100046641V3	ENSRNOT00000064279	4.138E-06	50.8	down	chr9	LOC108351994	453
ASRN6AP1B100024468V3	ENSRNOT00000042254	6.767E-06	41.1	down	chr7	Smgc	2779
ASRN6AP1B105980888V3	ENSRNOT00000086915	4.583E-09	27.6	down	chr12	Ache	2825
ASRN6AP1B105651172V3	ENSRNOT00000039383	8.259E-06	21.2	down	chr14	Cxcl13	1138
ASRN6AP1B108729299V3	ENSRNOT00000075728	5.7E-11	19.2	down	chr2	Col25a1	1443
ASRN6AP1B103518537V3	ENSRNOT00000038109	1.87E-11	16.0	down	chr7	Cyp11b2	1528
ASRN6AP1B100045840V3	ENSRNOT00000039564	3.087E-10	15.5	down	chr1	Lrtomt	877
ASRN6AP1B100060711V3	ENSRNOT00000049508	6.685E-06	15.5	down	chr3	LOC689220	693

Supplementary Figure S1. Secretory function of the submandibular gland of rats. The stimulated salivary flow rate of submandibular glands (μl/min). N=5/group, ** $P < 0.01$.

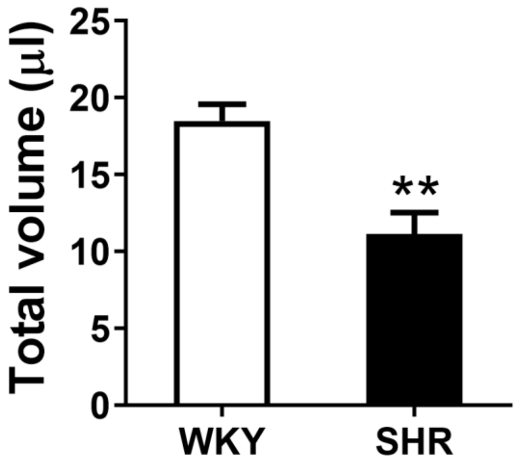

Supplementary Figure S2. Histological structure of submandibular glands in SHRs and WKY rats. (a) H&E staining of SMGs in SHRs, (b) H&E staining of SMGs in WKY rats. bar, 50μm.

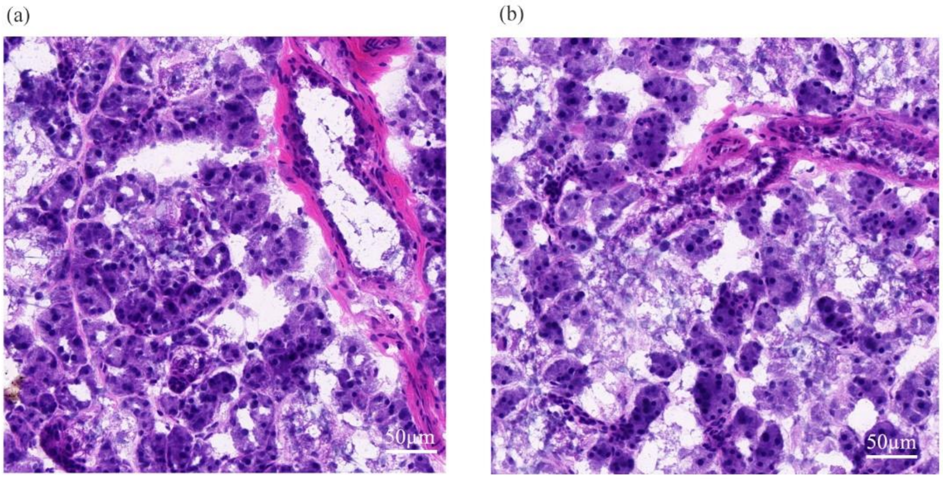

Supplement: Supplementary file 1 — Supplementary Information. [file 41598_2020_70853_MOESM1_ESM.pdf]
